# Supplementary material for: Collective production of hydrogen sulfide gas enables budding yeast lacking MET17 to overcome their metabolic defect
Source: PLoS Biol. 2023 Dec 7;21(12):e3002439. doi: 10.1371/journal.pbio.3002439 (PMC10729969; doi:10.1371/journal.pbio.3002439)
Supplement: S1 Figs — (PDF) [file pbio.3002439.s001.pdf]

S1 FIGURES

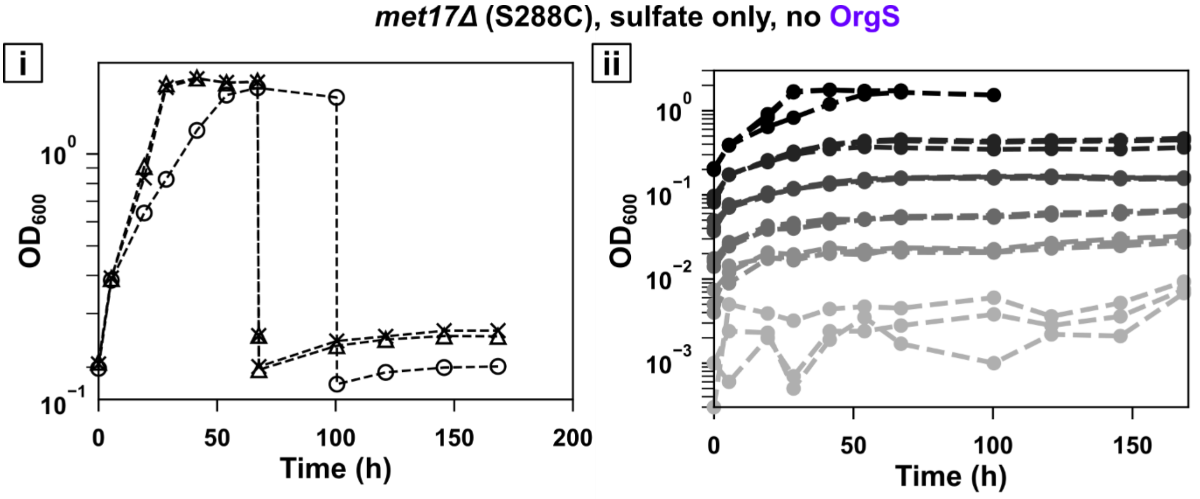

Figure A. *met17Δ* in the S288C background can grow on sulfate, but growth is less prominent than in the RM11 background. *met17Δ* (WY576, S288C) cells were grown in liquid SD minimal medium containing sulfate but no organosulfurs. Growth was assessed by measuring optical density at 600 nm (OD_{600}). (**i**) *met17Δ* can grow to saturation. Each line represents a technical replicate. Unlike in RM11, the saturated cultures did not regrow upon dilution. (**ii**) Growth of *met17Δ* on sulfate is density-dependent, but requires a high initial cell density to achieve saturation. Darker gray shades indicate higher initial cell densities, and three technical replicates were started at each density. Only cultures at the highest initial cell density tested (OD_{600} = 0.2) grew to saturation. Cultures in **i** are the same as the highest density cultures in **ii**. Cultures were of 7-ml volume in glass tubes of 18-mm diameter with loosely fitted plastic lids. The data underlying this figure can be found in S6 Data.

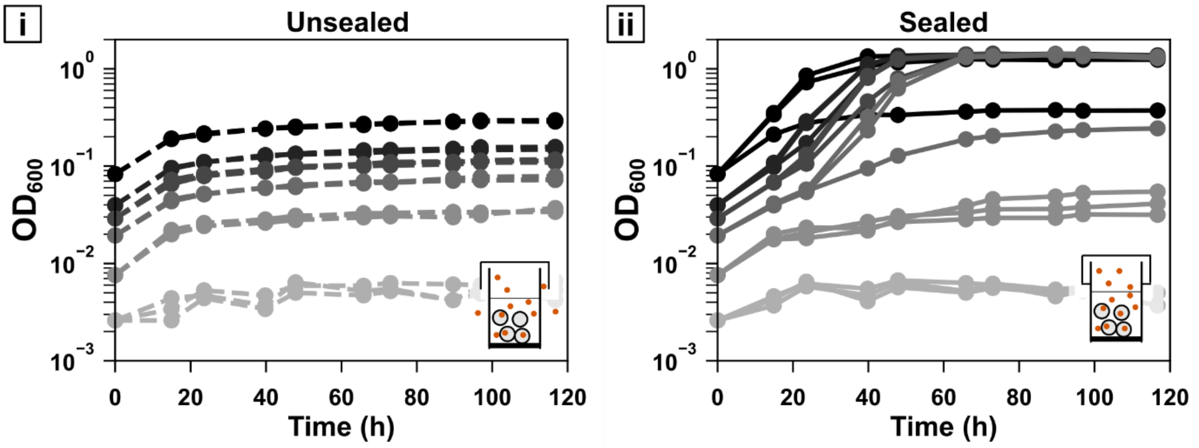

Figure B. Lowering gas escape increases the propensity of S288C *met17*Δ to grow on sulfate. *met17*Δ (WY576) cultures were initiated in SD medium at different initial cell densities (denoted by different shades of gray). At each density, 6 replicate cultures of 2.5 ml each were set up in glass tubes with loosely fitted plastic lids. Of these, 3 tubes were additionally sealed with parafilm to lower gas escape, while the remaining 3 only had plastic lids that allowed gas exchange. (**i**) Unsealed tubes showed only residual growth at all densities, unable to reach saturation. (**ii**) In contrast, in sealed tubes, growth was observed at initial cell densities of OD 0.02 and above, and stochasticity was evident as the three replicates at a given initial cell density showed divergent growth dynamics. The data underlying this figure can be found in S7 Data.

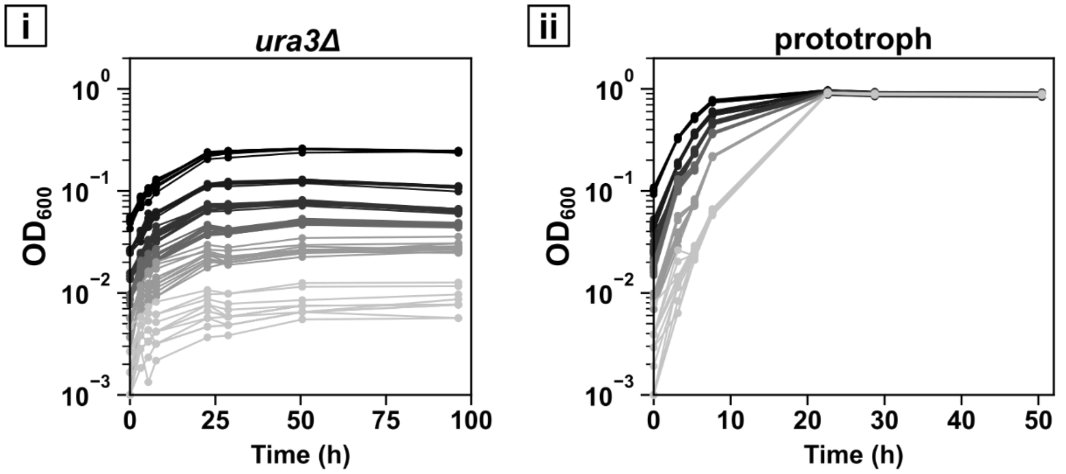

Figure C. Sharing headspace between different cell densities does not alter the growth dynamics of uracil auxotrophs and prototrophs. To test if exchange of gaseous metabolites impacts the growth of other auxotrophs or of prototrophs, cultures of each genotype were initiated at different cell densities in SD medium in 96-well plates (where headspace is shared). Darker gray shades denote higher initial cell densities. (**i**) Uracil auxotrophs *ura3*Δ (WY1318) could not grow at any initial cell density, which was consistent with observations made in glass tubes where different densities did not share headspace (Fig 1D, right panel). *ura3*Δ yeast were grown to exponential phase in SD medium supplemented with uracil before being washed in SD medium and diluted to different densities. (**ii**) All cell densities of the prototroph (WY1870) immediately started growing and grew to saturation within 20 hours, which was also consistent with observations in glass tubes (Fig 1D, left panel). Prototrophs were grown to exponential phase in SD medium before being diluted to different cell densities. Population growth was assessed by measuring OD_{600} in a plate reader. Plates were sealed with plastic lids reinforced with parafilm. Strains are of RM11 background. The data underlying this figure can be found in S8 Data.

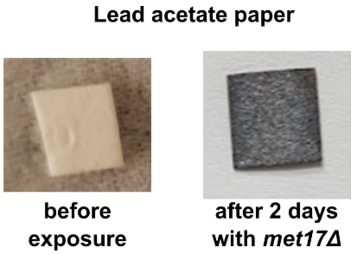

Figure D. Hydrogen sulfide gas is released by *met17*Δ growing on sulfate. A square of lead acetate paper placed in one of the gaps between the wells of a 96-well plate where *met17*Δ were growing in SD medium turned black after 2 days. Black colour indicates the formation of lead sulfide upon reaction of lead acetate with hydrogen sulfide. Strain: WY2531.

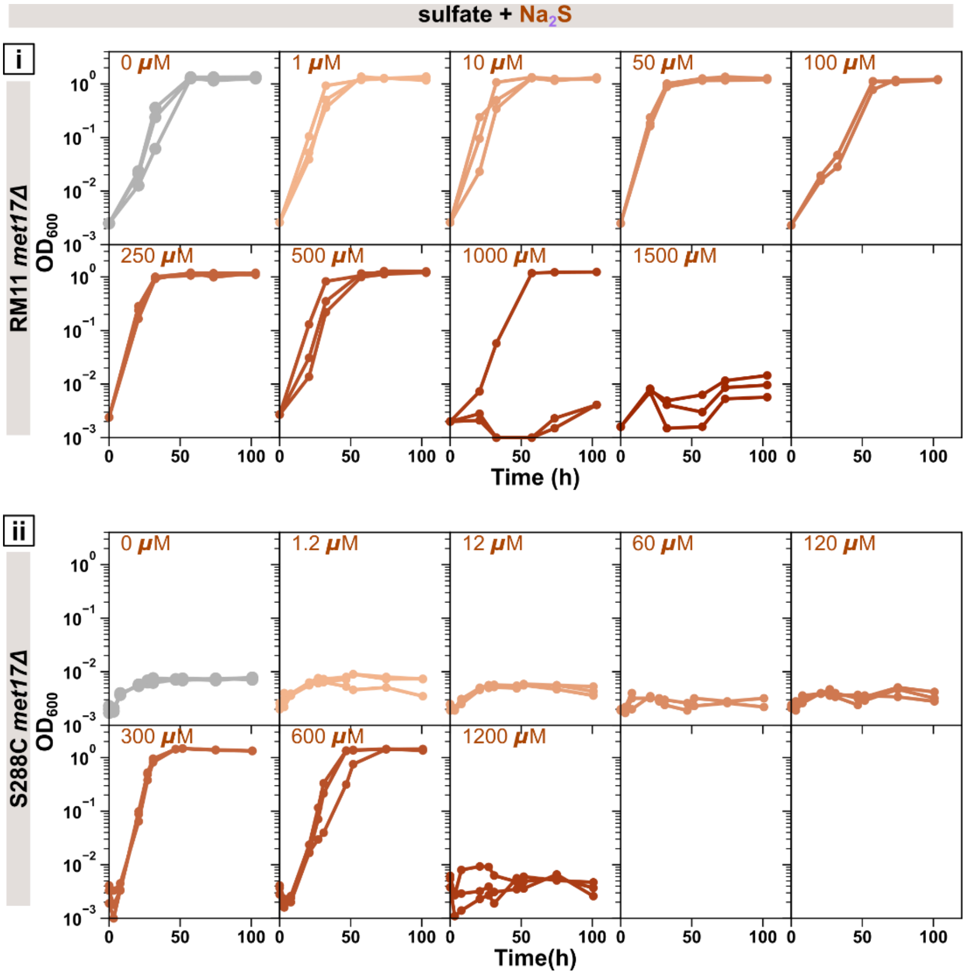

Figure E. Sodium sulfide promotes the growth of *met17*Δ in sulfate over a range of concentrations but becomes toxic at high concentrations. Different concentrations of sodium sulfide (Na_2S) were added to low-density cultures of *met17*Δ in SD medium. Three technical replicates were set up for each sulfide concentration and tubes were additionally sealed with parafilm and cling film to prevent the loss of sulfide. (**i**) Growth curves of RM11 *met17*Δ (WY2548). Note that even without additional sulfide, the cultures grew to saturation because the additional sealing reduced escape of sulfide released by the cells (top left in **i**, gray lines). Addition of up to 500 μM of Na_2S sped up cell growth, i.e. growth curves reached saturation at an earlier timepoint than in the absence of additional Na_2S (gray). The curves at 100 μM were an exception that showed slower growth, but this was not a consistent observation. Concentrations of 1 mM and above could inhibit growth. Culture volume was 3 ml in 13-mm glass tubes. (**ii**) Growth curves of S288C *met17*Δ (WY2590). Growth promotion of *met17*Δ on sulfate was only observed with addition of 300 or 600 μM Na_2S. The highest concentration tested (1.2 mM Na_2S) impaired growth. Cultures were set in 13-mm glass tubes, at a volume of 3 ml in **i** and 2.5 ml in **ii**. Thus, corresponding sub-panels in **i** and **ii** involved equal amounts (nmoles) of sulfide, though the concentrations differ. The data underlying this figure can be found in S9 Data.

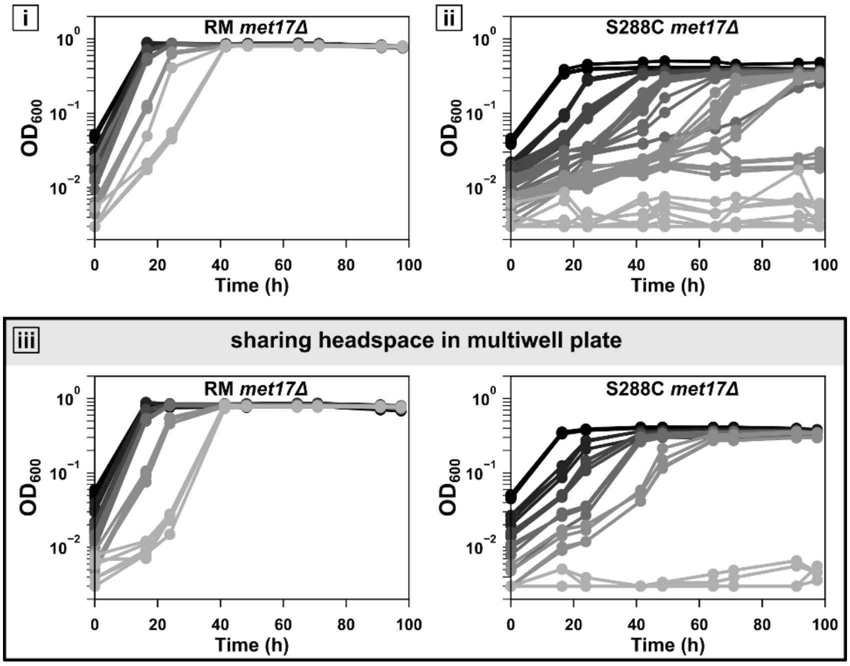

Figure F. S288C *met17*Δ release lower H_2S than RM11 *met17*Δ. Different initial cell densities of *met17*Δ were grown in liquid minimal medium in separate wells of a 96-well plate, thus sharing headspace. (**i**) All densities of RM11 *met17*Δ (WY2531) grew to saturation within 40 hours. (**ii**) In contrast, S288C *met17*Δ (WY2515) required much longer times to grow to saturation, and lower cell densities never grew to saturation. (**iii**) When the two strains occupied different wells of the same 96-well plate such that they shared headspace and volatiles, all except the lowest cell density of S288C *met17*Δ grew faster (compare with **ii**). Since growth propensity increases with increasing sulfide concentrations, the growth promotion of S288C in the vicinity of RM11 indicates that RM11 *met17*Δ release more H_2S than S288C *met17*Δ. For the S288C strain, SD medium was additionally supplemented with histidine, leucine, and uracil to cover the nutritional requirements caused by additional (engineered) auxotrophic mutations. The data underlying this figure can be found in S10 Data.

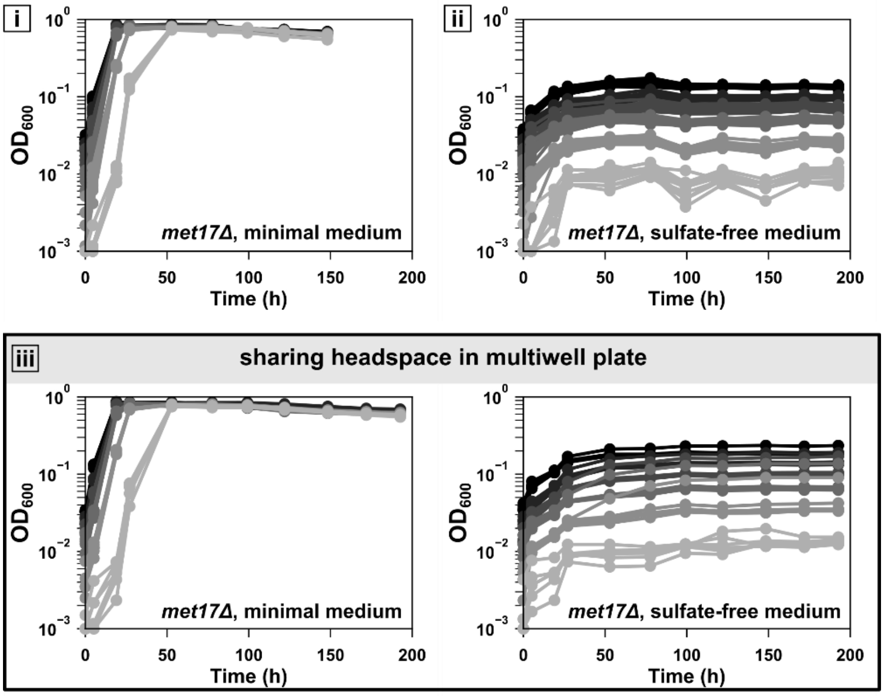

Figure G. Growth of *met17*Δ in minimal medium requires inorganic sulfate. While all densities of *met17*Δ (WY2548, RM11) could grow to saturation in minimal medium containing sulfate (SD) when sharing headspace in 96-well plates (**i**), *met17*Δ could not grow in sulfate-free medium at any cell density (**ii**). (**iii**) Even when these two sets of populations shared headspace in the same 96-well plate, *met17*Δ could not grow in sulfate-free medium, suggesting that sulfide from neighbouring wells is insufficient to support the growth of *met17*Δ and they require the sulfide they themselves produce by sulfate reduction. This is in contrast to *met14*Δ that are unable to produce their own sulfide but could grow on sulfide from neighbouring wells (Fig 2E). One possible explanation is that while *met17*Δ can grow by assimilating sulfide, their mechanism of utilizing sulfide is of low efficiency compared to the Met17-dependent sulfide assimilation observed in *met14*Δ. The data underlying this figure can be found in S11 Data.

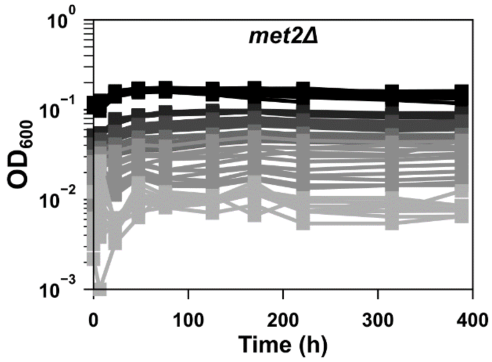

Figure H. *S. cerevisiae* cannot bypass the need for *MET2* to assimilate inorganic sulfate. *met2*Δ (WY2538, RM11) could not grow in SD medium at any initial cell density. Higher initial cell densities are denoted by darker shades of gray. All populations were sharing headspace in a 96-well plate to favour the exchange of H_2S and maximize the chances of growth by a sulfide-dependent alternative mechanism. The data underlying this figure can be found in S12 Data.

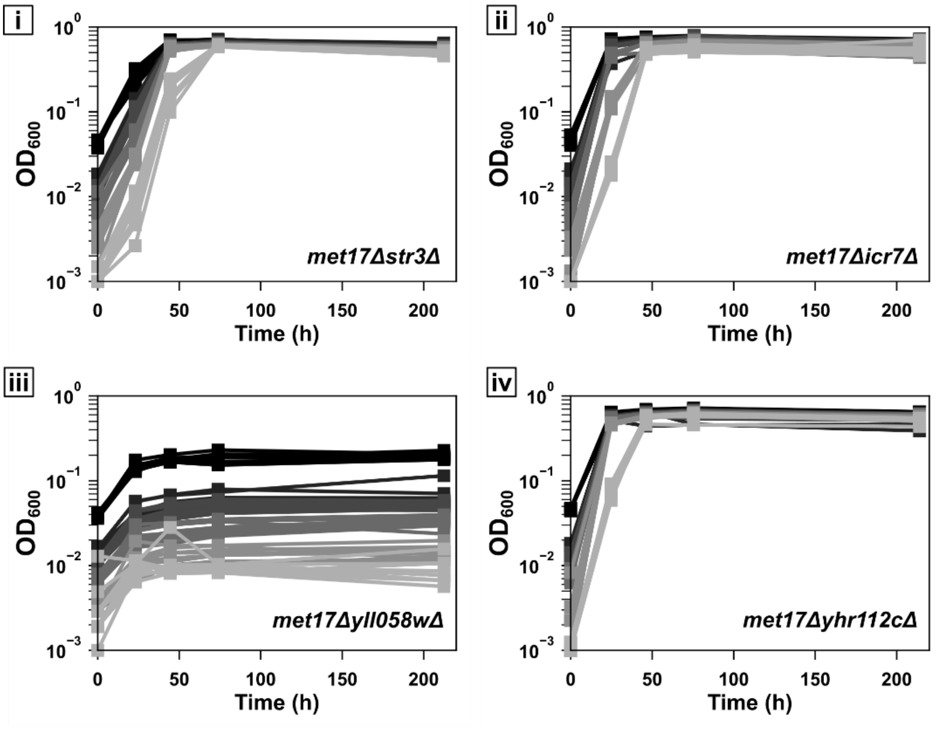

Figure I. Identifying the gene responsible for assimilating sulfide in *met17*Δ. Four candidates, selected on the basis of sequence similarity to Met17, were tested through the double mutant screen described in Fig 3B. In brief, we were looking for the gene which when deleted along with *MET17* completely abrogated growth on sulfate (i.e. in minimal medium without organosulfur supplements). Strains belonged to the BY4741 (S288C) Yeast Deletion Library which, along with a *met17* deletion, also lacked synthesis of histidine, leucine and uracil. SD minimal medium was thus supplemented with these three nutrients, but methionine was removed once the cells grew to exponential phase. Cultures were diluted to different cell densities (denoted by different shades of gray) and grown in a 96-well plate to maximize the chances of sulfide-dependent population growth of *met17*Δ. Of the candidates tested, only *YLL058W* passed the screen with only residual growth observed at all cell densities (**iii**). Strains were WY2584 (*str3*Δ) in (**i**), WY2587 (*icr7*Δ) in (**ii**), WY2586 (*yll058w*Δ) in (**iii**) and WY2585 (*yhr112c*Δ) in (**iv**). The data underlying this figure can be found in S13 Data.

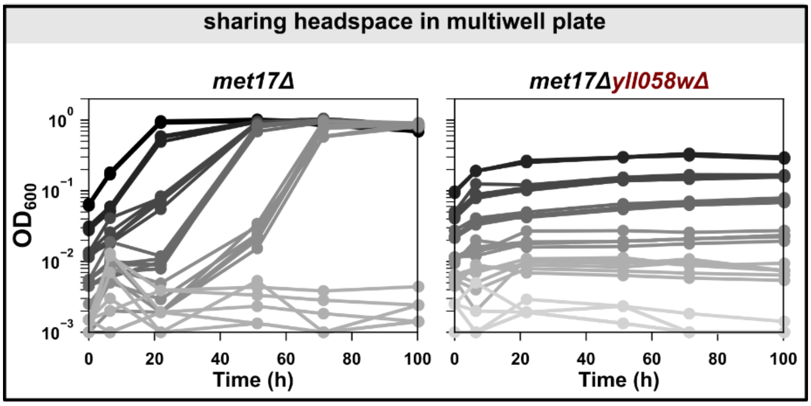

Figure J. Sulfide assimilation is defective in *met17*Δ*yll058w*Δ double knockouts. Double mutants *met17*Δ*yll058w*Δ (WY2595) were inoculated in SD medium at different initial cell densities in the same 96-well plate where different densities of *met17*Δ (WY2590) were growing. This should allow *met17*Δ*yll058w*Δ to consume H_2S released by *met17*Δ, as was observed for *met14*Δ in Fig 2E. However, the double mutant could not grow indicating that they are impaired in sulfide assimilation even when sulfide was supplied. Both strains are of S288C background. The data underlying this figure can be found in S14 Data.

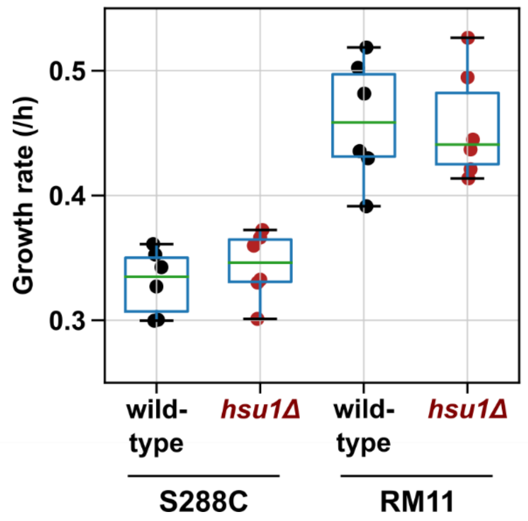

Figure K. Exponential growth rates of *wildtype* and *hsu1*Δ are comparable. Growth rates of wildtype and *hsu1*Δ yeast during exponential growth in SD medium are not significantly different (p-value of 0.19 for S288C and 0.83 for RM11, two-tailed paired Student's t-test, where growth rates measured on the same day were paired). Growth rates were calculated as the slope of natural log of optical density measurements against time. Box plot shows quartiles, with whiskers extending to the most extreme datapoints and median marked as the green line. S288C wildtype strains were WY1364, WY1376, WY1377, while *hsu1*Δ were WY2608, WY2612, WY2637. RM11 wildtype strains were WY1810 and WY1870, while *hsu1*Δ were WY2643, WY2640 and WY2652. The data underlying this plot can be found in S15 Data.

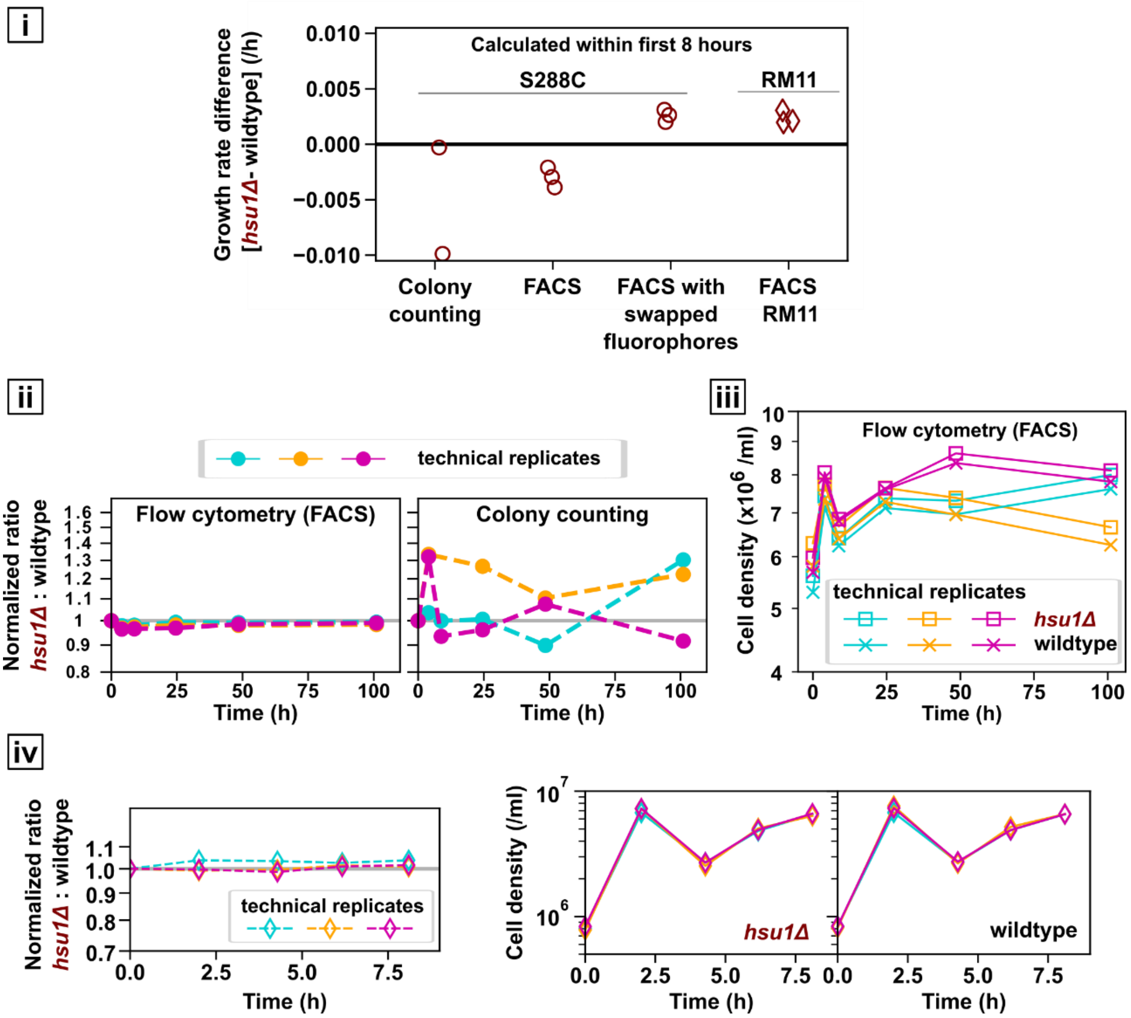

Figure L. No disadvantage detectable for *hsu1*Δ under sulfur starvation. (**i**) Competition assays between wildtype and *hsu1*Δ under sulfur starvation show high variation in outcome and no trend could be detected in the growth rate difference between the genotypes. Growth rate difference between *hsu1*Δ and wildtype was measured as the slope of natural log of ratios in co-cultures over the first 8 hours. Ratios were determined either by colony counting or by flow cytometry (FACS). For a given condition, each datapoint represents a technical replicate. For S288C, swapping fluorophores on the genotypes led to a small positive deviation, whereas the original pairing produced a small negative deviation, suggesting a minor contribution from such experimental details. Overall, the fitness difference between wildtype and *hsu1*Δ, if any, was too small to be detected in our assay. S288C strains were WY2637 (*hsu1*Δ) against WY1377 (wildtype) in the first two conditions, and WY2612 (*hsu1*Δ) and WY1364 (wildtype) for fluorophore swapping. RM11 strains were WY2643 (*hsu1*Δ) against WY1870 wildtype). Ratio (**ii**) and cell density (**iii**) dynamics from the competition assays between S288C wildtype (WY1377) and *hsu1*Δ (WY2637) under sulfur starvation. The two genotypes were mixed at a 1:1 ratio in sulfate-free medium and population dynamics of 3 technical replicates were followed using both flow cytometry and colony counting at each timepoint. Samples were appropriately diluted to get single events on flow cytometry and for manually counting colony forming units on a rich medium plate. Each genotype carried a distinct fluorescent protein to allow identifying the population. Ratios were normalized to initial value. The ratio remained largely stable in flow cytometry data (**ii**, left) whereas large variability with no distinct trend was observed in colony counting (**ii**, right). Colony counting is inherently noisy as counting can only be done on the order of hundreds, whereas flow cytometry measures tens of thousands of events at each time point. Population dynamics measured by flow cytometry (**iii**) shows that although there were variations among tubes, within a tube the population sizes of two genotypes were very similar. The initial residual growth was enabled by stored cellular sulfur. (**iv**) Ratio (left) and cell density (right pair) dynamics from FACS-based competition assays between RM11 *hsu1*Δ (WY2643) and wildtype (WY1870) during sulfur starvation. Ratios deviate very little from 1 (horizontal gray line) over the 8-hour period, and the slope of each trendline was plotted as the growth rate difference in **i** (diamonds). The pair of graphs on the right show cell density of each genotype over time, with the trendlines from all replicates largely overlapping. The data underlying this figure can be found in S16 Data.

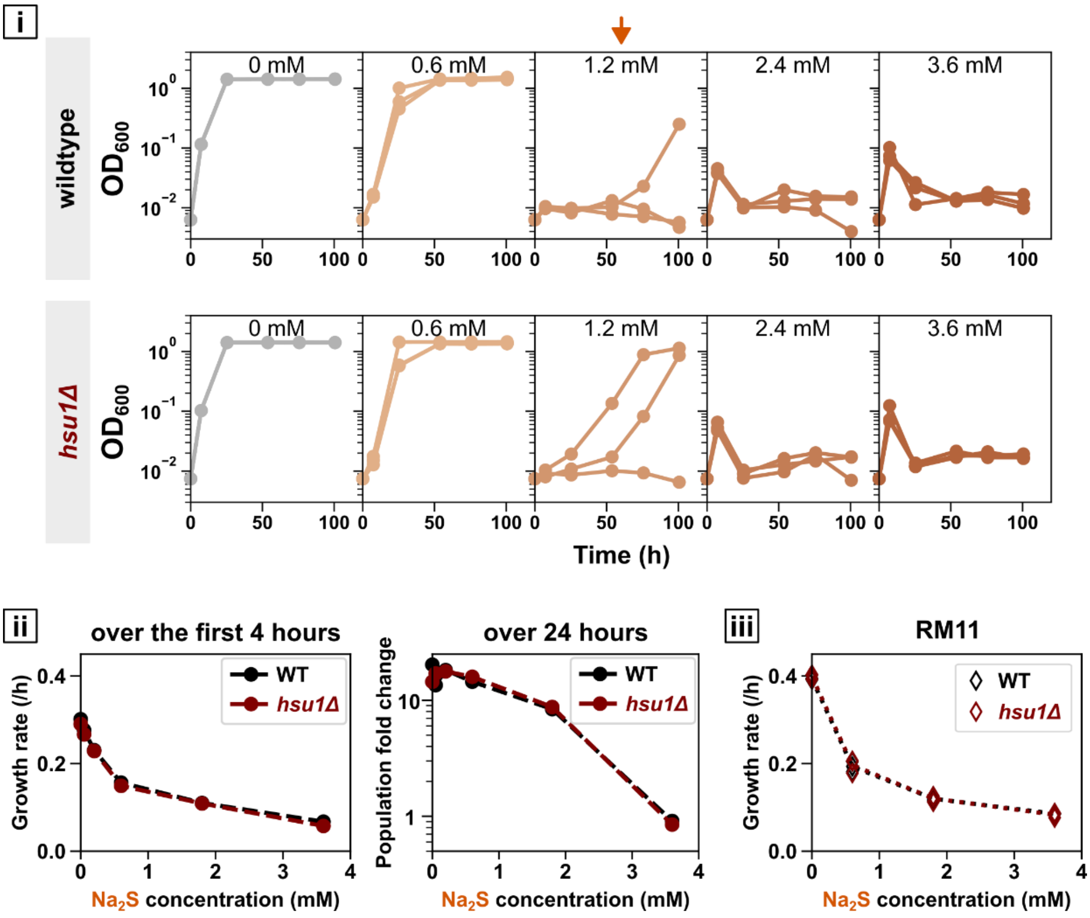

Figure M. *hsu1*Δ and wildtype behave similarly under high sulfide exposure. (**i**) The concentration where sulfide impairs growth is similar for *hsu1*Δ (WY2597) and wildtype (WY2601) populations. The effects of different concentrations of Na_2S on yeast growing in SD minimal medium were compared by measuring population dynamics using optical density. For both genotypes, at 1.2 mM sulfide, at least one of three replicate populations failed to grow (highlighted with orange arrow). Strains are of S288C background. (**ii**) Sulfide exposure impacts the growth rate (left panel) and population dynamics (right panel) of *hsu1*Δ (WY2637) and wildtype (WT; WY1364) to a similar extent. Growth rate was measured as the slope of natural log of OD measurements over the first four hours of sulfide exposure in 2.5-ml SD medium cultures. Increasing concentrations of Na_2S led to a decrease in growth rate in both genotypes. Population fold change (right panel) was estimated by scaling the population size determined by flow cytometry after 1 day of growth in different concentrations of Na_2S to the population size before adding Na_2S. Viability of cells was separately confirmed by colony counting. Both datasets in B come from the same experiment with S288C strains. (**iii**) In RM11 background, sulfide exposure impacts the growth rates of both *hsu1*Δ (WY2640) and wildtype (WT; WY1870) to a similar extent. For each genotype, a datapoint represents the growth rate estimated from OD measurements of one of three replicate 2.5-ml monocultures. Dotted lines connect means at each Na_2S concentration. The growth rates of the two genotypes did not differ significantly at any Na_2S concentration tested (using two-tailed t-tests with a p-value threshold of 0.05). The data underlying this figure can be found in S17 Data.

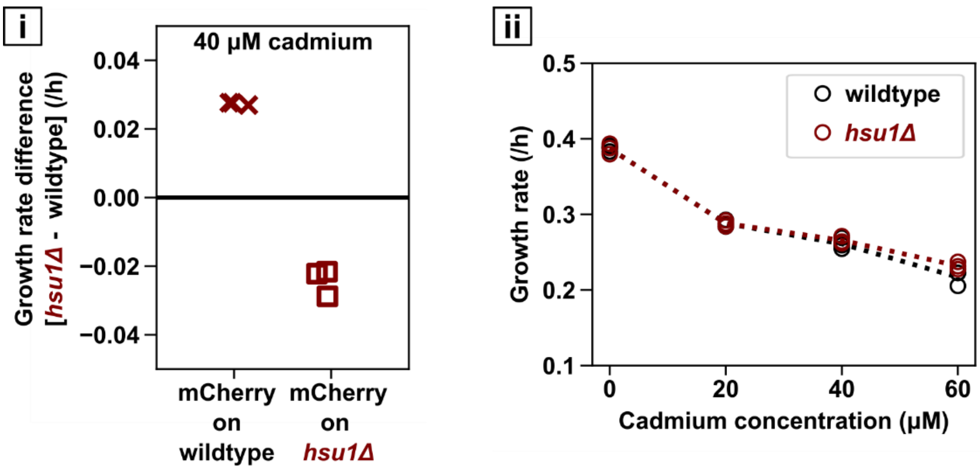

Figure N. *HSU1* provides no detectable advantage under cadmium exposure. (**i**) Cadmium tolerance of *hsu1*Δ and wildtype yeast could not be assessed by co-culture competition experiments as the choice of fluorophore had a dramatic impact on the outcome. Datapoints represent the extent of deviation of the growth rate of *hsu1*Δ from wildtype (horizontal black line). Each symbol represents an independent competition assay (same as in Fig 4E), with 3 technical replicates each. The fluorescent proteins that the two genotypes expressed for flow cytometric detection were swapped between crosses and squares, leading to a diametrically opposite measurement of growth rate difference. This may result from cadmium affecting the autofluorescence of the yeast. Crosses used strains WY2652 (*hsu1*Δ*)* against WY1870 (wildtype), while squares used WY2640 (*hsu1*Δ) against WY1810 (wildtype). The co-cultures were carried out in liquid SD medium with 40 μM cadmium sulfate. (**ii**) Cadmium treatment impairs the growth rates of wildtype (WY1870) and *hsu1*Δ (WY2640) to a similar extent. Different concentrations of cadmium sulfate were added to 3-ml monocultures of each genotype in 3 replicates, and growth rates were estimated as slope of natural log of OD measurements over time. Dotted line connects mean growth rates of a genotype at different cadmium concentrations. The growth rates of the two genotypes did not differ significantly at any cadmium concentration (using two-tailed t-tests with a p-value threshold of 0.05). The data underlying this figure can be found in S18 Data.

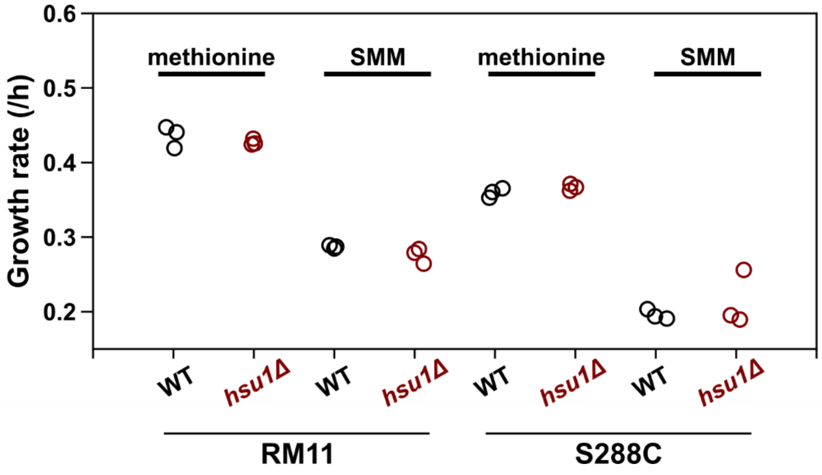

Figure O. *hsu1*Δ and wildtype show a similar growth rate on methionine and S-methylmethionine. The growth rates of *hsu1*Δ and wildtype (WT) were comparable in sulfate-free medium supplemented with either methionine or S-methylmethionine (SMM). Both genotypes in both strain backgrounds had a slower growth rate on SMM as compared to methionine. Cells growing exponentially in SD medium were washed with sulfate-free medium and starved for 24 hours before adding either methionine or SMM. Growth was assessed by OD measurements and once cells were growing exponentially after the initial lag phase. Growth rates were calculated as the slope of natural log of OD for each of 3 technical replicates. RM11 strains were WY2639 (*hsu1*Δ) and WY1870 (wildtype), while S288C strains were WY2597 (*hsu1*Δ) and WY2601 (wildtype). The growth rates of the two genotypes did not differ significantly on either organosulfur in either background (using two-tailed t-tests with a p-value threshold of 0.05). The data underlying this figure can be found in S19 Data.

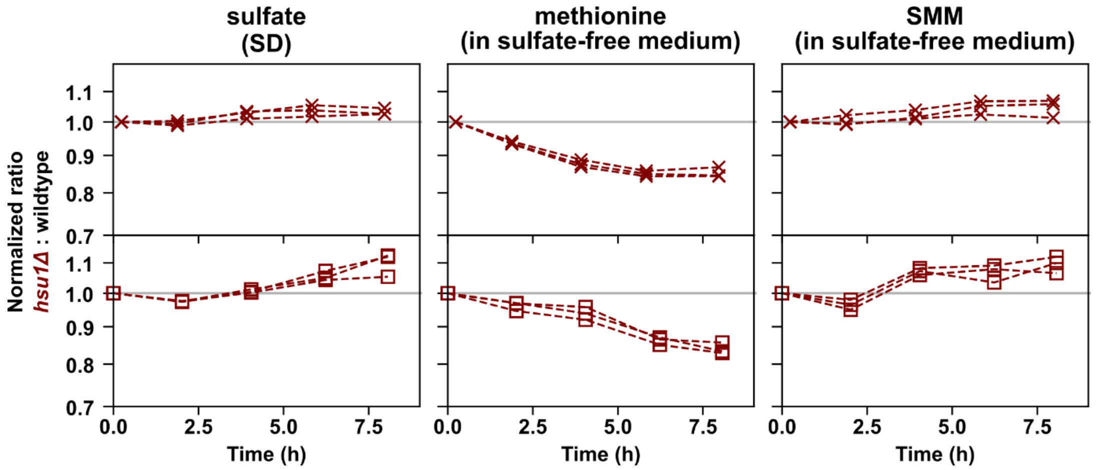

Figure P. Competition assays in RM11 yeast reveal a pleiotropic role of *HSU1* in organosulfur assimilation. The dynamics of genotype ratios from competition assays show a consistent fitness disadvantage of *hsu1*Δ in using methionine as the sole sulfur source, and a slight advantage in using SMM and sulfate. Trendlines are from two competition assays (crosses and squares) with 3 technical replicates each. Strains were grown to exponential in the relevant sulfur environment in monocultures, before being diluted to OD_{600} 0.7 in the same medium and combined in a 1:1 ratio. Strain ratios were assessed over time by flow cytometry. Cells showed exponential growth followed by saturation under all three conditions. The difference in growth rate of the genotypes (Fig 4E, crosses and squares) was calculated from these datapoints using only the exponential phase of growth, which corresponds to $OD_{600} < 0.5$ on our set-up. The fluorescent proteins expressed by the genotypes were swapped between the two competition assays, thus controlling for any bias arising from fluorophore choice. Crosses used strains WY2652 (*hsu1*Δ*)* and WY1870 (*HSU1*), while squares used WY2640 (*hsu1*Δ) and WY1810 (*HSU1*). The data underlying this figure can be found in S20 Data.

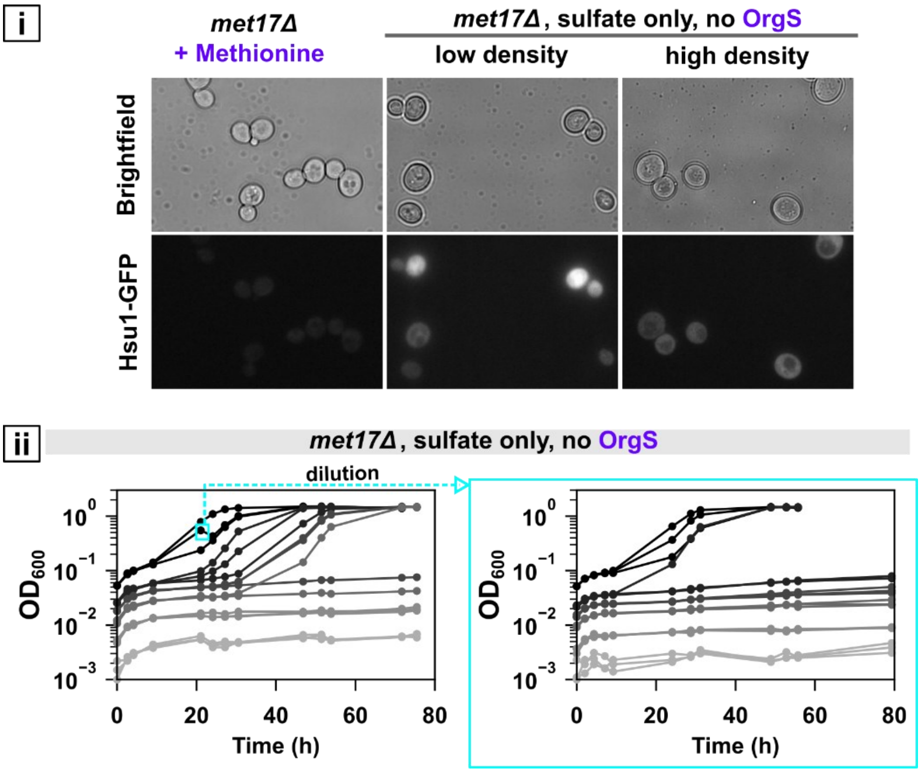

Supplementary Figure Q: A cell-state switch does not underpin the stochastic growth dynamics of S288C *met17*Δ in sulfate. (**i**) The expression of *HSU1-GFP* in S288C *met17*Δ (WY2620) does not correlate with growth status. While exponential cells growing in methionine do not show considerable expression of Hsu1-GFP (left column), cultures inoculated at both low and high cell densities show expression within 4 hours of transfer to SD minimal medium containing sulfate, but no organosulfurs. These cultures did not eventually grow to saturation, indicating that if a cell-state switch results in the stochastic growth dynamics, the expression of Hsu1-GFP cannot be the mechanism of the switch. (**ii**) If a cell-state switch exists, *met17*Δ that have started to grow on sulfate (cyan rectangle in the left panel) should already have switched on. If density-dependence relies on the cell-state switch, such cells should grow even at low-cell densities when re-inoculated into SD medium (right panel). However, this was not observed, suggesting that density-dependence does not result from a cell-state switch or that the switch is too transient to persist through the dilution. The data underlying these plots can be found in S21 Data. Strain: WY2590.

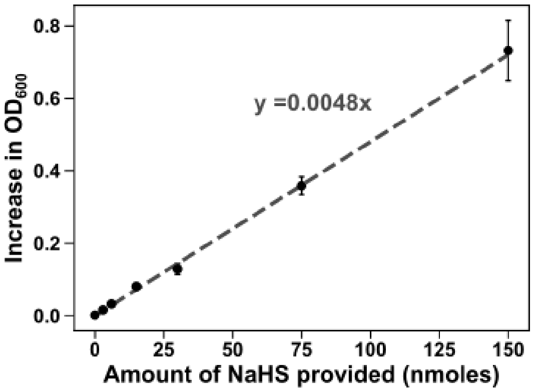

Supplementary Figure R: Consumption rate of sulfide c is approximately 3 fmole/cell birth in RM11 yeast. RM11 *met14*Δ yeast (WY2539) were grown to exponential phase in SD medium supplemented with methionine and washed and transferred to sulfate-free medium. Cells were starved for 24 hours before addition of different amounts of sodium hydrosulfide (NaHS) to each of the three replicate cultures. An end-point optical density measurement was done after 3 days and the increase in OD_{600} was plotted against the amount of NaHS provided. The slope of the linear regression (0.0048 /nmole) was used to calculate the amount of sulfide consumed per cell birth. 1 unit of OD_{600} corresponds to $7x10^7$ cells in our set-up. The data underlying this plot can be found in S22 Data.

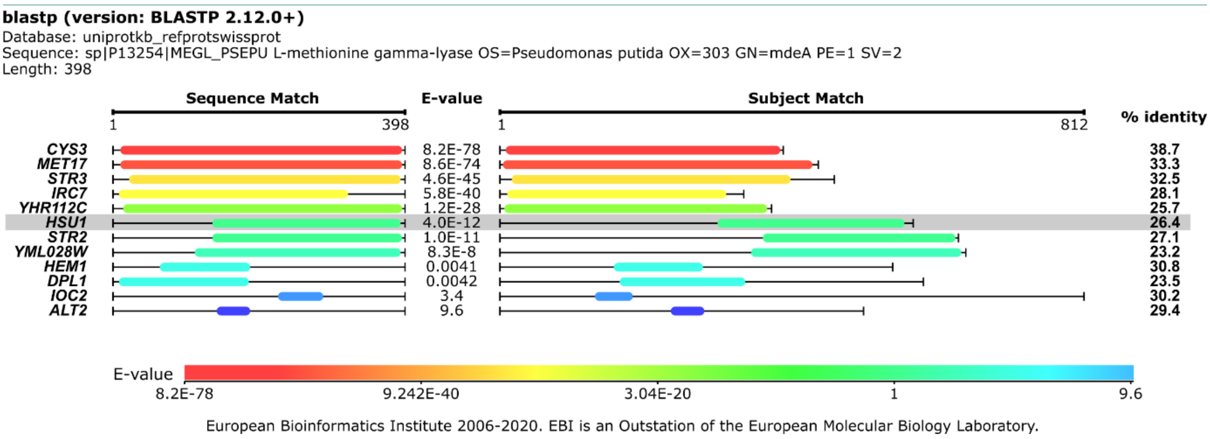

Supplementary Figure S: *HSU1* shares homology with L-methionine gamma-lyase from *Pseudomonas putida.* A protein-sequence BLAST of the methionine gamma-lyase MdeA from *P. putida* (UniProt ID P13254) against the *S. cerevisiae* genome picks up multiple sulfur metabolism genes. *HSU1* (highlighted in gray) shares 26% identity and a high expectation value with the reference sequence. Graphics generated from UniProt.

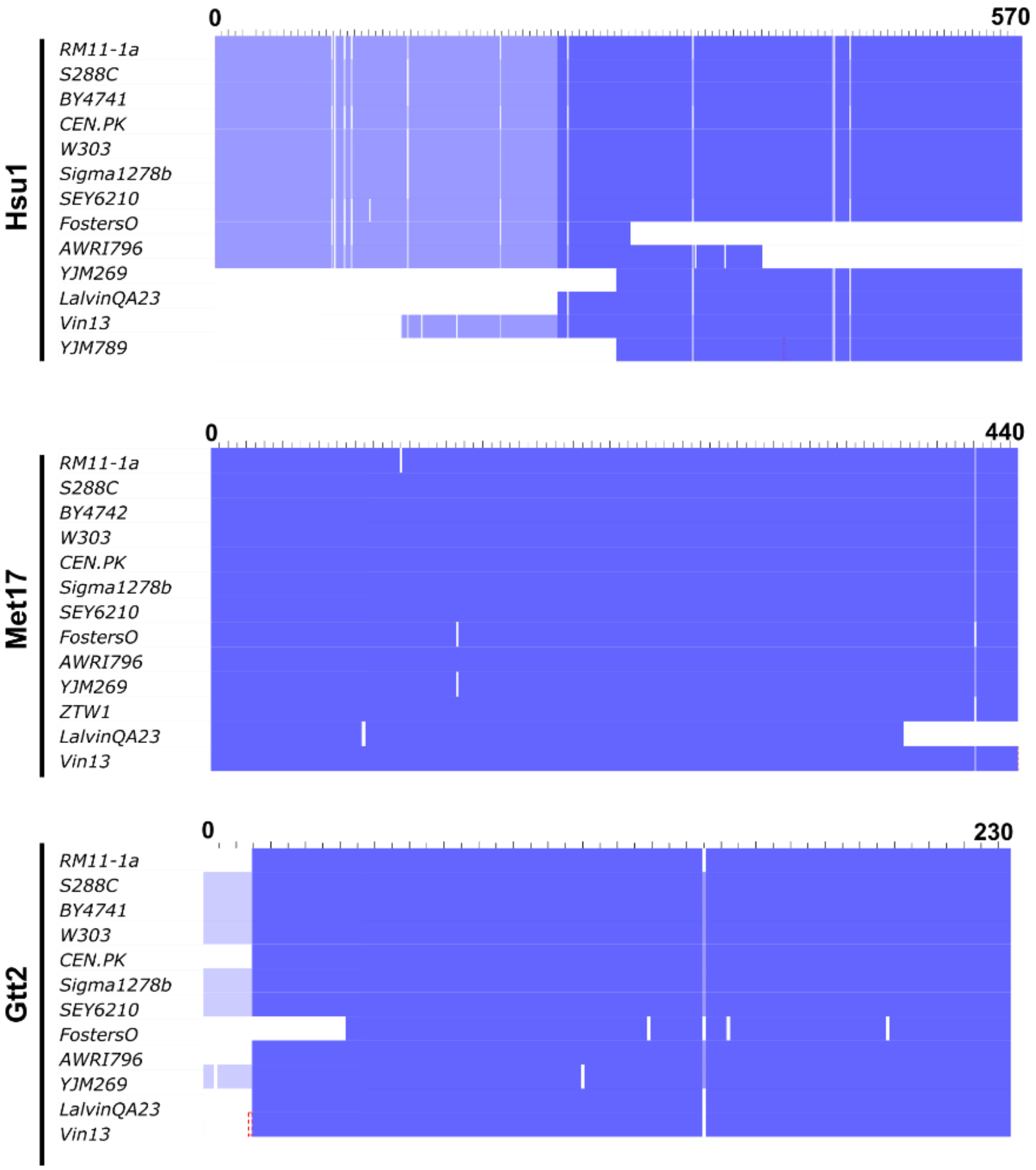

Supplementary Figure T: Hsu1 protein shows a high degree of polymorphism among yeast strains. Protein sequences from 41 strains in the *S. cerevisiae* Genome Database were aligned for the protein Hsu1, Met17 and Gtt2. A higher percentage of identity is denoted by darker blue shades. A selection of the strains is shown here to highlight the high degree of polymorphisms tolerated in Hsu1, compared to Met17. Notably, large deletions were observed in Hsu1 sequences from some strains. *HSU1* is located close to the telomere on Chromosome XII. However, *GTT2* which is more proximal to the telomere shows fewer regions of variability. Variations in the initial stretch may result from erroneous annotation of start codon. Altogether, these data suggest that the contribution of *HSU1* to the cell's fitness may be more variable than that of Met17 or Gtt2.
