# Supplementary material for: Collective production of hydrogen sulfide gas enables budding yeast lacking MET17 to overcome their metabolic defect
Source: PLoS Biol. 2023 Dec 7;21(12):e3002439. doi: 10.1371/journal.pbio.3002439 (PMC10729969; doi:10.1371/journal.pbio.3002439)
Supplement: Attachment — Submitted filename: Response_to_reviewers.pdf [file pbio.3002439.s028.pdf]

RESPONSE TO REVIEWERS

NOTE: Reviewer's comments are in blue, and our responses are in black.

Reviewer #1: The paper by Sonal et al. adds to the recent discovery that yeast cells lacking MET17 can grow on sulfate when the cell number is high. At those conditions, the buildup of hydrogen sulfide in met17 mutants is used by Hsu1, and sulfur is assimilated. Overall, the authors do a good job of describing these mutants' growth in various conditions and conclude that the efficiency of using the accumulated hydrogen sulfide is low, and the reaction happens only at high cell densities. The results reinforce each other in the context of the other recently published papers on the same topic.

We thank the reviewer for a succinct summary of our findings.

Some points that need to be considered:

- Overall, the central unanswered question now is the putative function of Hsu1 in wild-type cells since the authors find no fitness advantage that HSU1 confers to wild-type yeast cells (not met17 mutants) during sulfur starvation. They speculate on its role during some sulfur-related stress responses. This should be tested experimentally. Upon exposure to cadmium, which triggers a well-described increase in glutathione synthesis, what is the viability of cells lacking or over-expressing HSU1? Additional stress conditions they mention (e.g., heat) could also be tested. Providing these answers is significant since Yu et al. reported some fitness effects attributed to Hsu1, which were not seen by Sonal et al.

We agree that the function of *HSU1* remains the one unresolved issue in this story. We followed the reviewer's advice as well as our own hunches to test some additional conditions during the review process. Cadmium exposure negatively impacted the growth rate of wildtype and *hsu1Δ* yeast to a similar degree (S14B Fig). We attempted to use a highly sensitive flow cytometry-based competition assay to discern the fitness effect of *hsu1* deletion during cadmium exposure. Unfortunately, cadmium stress appeared to interfere with the fluorescent properties of the cells, and we could not detect an effect beyond the bias introduced by the choice of fluorophore (S14A).

Interestingly, we detected a fitness effect when we competed wildtype and *hsu1Δ* yeast during exponential growth on different sulfur sources—sulfate, methionine and S-methylmethionine (SMM, an organosulfur which yeast usually obtain from plant sources). *HSU1* confers an advantage during growth on methionine, but a mild disadvantage during growth on either SMM or sulfate (Fig 4E). As previously mentioned in the Discussion, our assays have much higher statistical power compared to the high-variance colony counting methodology used by Yu *et al*. We have also carried out the appropriate control of swapping fluorophores on the genotypes. We thus have high confidence in this result. We have updated the Discussion to reflect this pleiotropic role of *HSU1* in the assimilation of different sulfur compounds (see Discussion: "Possible functions of *HSU1* in sulfur metabolism"). We provide reasoning to speculate that *HSU1* may belong to a class of multicatalytic sulfur metabolism enzymes. While testing this hypothesis would require extensive biochemical and metabolomics analyses, which are beyond the scope of the current study, we hope that the reviewer will agree that these new findings add value to the manuscript by shedding light on *HSU1*'s function.

- Can over-expression of Hsu1 shorten the lag in the growth of met17 mutants? In the model, they propose that Hsu1 is not efficient and works only at high sulfide concentrations. One might expect that providing more Hsu1 would increase the reaction rate, in a largely linear manner.

We have added additional experiments to address this suggestion (Fig 3E). As the reviewer anticipated, overexpression of *HSU1* indeed shortened lag times at lower cell densities of *met17Δ*. To make our work comparable with Van Oss *et al.*, we used plasmids from their lab. That is, we overexpressed *HSU1* from plasmids and, since plasmid-carrying strains grew slower than those without plasmids, we compare lag times with a control strain where the plasmid carries a catalytically defective *HSU1* mutant. Because the promoter is constitutive, we cannot test a linear relationship between *HSU1* expression and lag times. Such titration experiments would be challenging: Lag times are sensitive to the exact initial cell densities used in the experiment as well as to day-to-day variation in growth conditions. For small variation in lag time, this assay would require a high number of replicates.

Reviewer #2: In "When is an auxotroph not an auxotroph: how budding yeast lacking MET17 collectively overcome their metabolic defect" Sonal et al. describe and try to explain a leaky auxotrophy in met17_delta yeast strains. There is novelty and value to this manuscript, especially for the functional study of the HSU1 ORF and for the wine industry. However, there are some major issues that I would like to see addressed before recommending it for publication. Below are my main criticisms.

Throughout the study the growth behaviour of various strains from deletion collections are studied. However, the omission of positive and negative controls (such as a prototrophic and a "true auxotrophic" strain, respectively) in each experiment makes it impossible to assess the effects of each perturbation. Examples include Figures 2, 3 and 5. This is a critical point for me.

We thank the reviewer for this suggestion as we feel that orienting our readers to the expected growth curves for prototrophs and true auxotrophs could make our article accessible to a broader audience. We have therefore added graphs to show equivalent growth curves and images of patching on agar plates for a prototrophic strain as well as an unrelated auxotroph (*ura3Δ*) in Fig 1. As expected, the prototroph grows without lags at all densities and the uracil auxotroph does not grow at any density (Fig 1D). Both growth behaviours are strikingly distinct from the density-dependent growth of *met17Δ.* Corresponding to growth curves of *met17Δ* in 96-well plates where headspace is shared (Fig 2C), we have added growth curves for the prototroph and the uracil auxotroph in the same growth conditions (S3 Fig). These growth curves also serve as controls for Fig 3C, where we claim that *hsu1Δ* behave as prototrophs, while *met17Δhsu1Δ* behave as true auxotrophs.

Note, however, that the remaining datasets rely on the density-dependent growth of *met17Δ* (e.g., the effect of extraneous sulfide on lag times and stochasticity in Fig 2G, and persistence of density-dependence upon re-dilution in Fig 5C). These claims cannot use strains that show no density-dependence as controls. We assert however that we have included any controls needed to support our claims in all datasets. Where growth at lower densities was shown to be enhanced by adding sulfide, we compare to growth curves where no extraneous sulfide was provided (Fig 2G). Where *HSU1* expression is reported in organosulfur-starved *met17Δ*, we compare with *met17Δ* growing on methionine (Fig 5B).

It is well known that carbon dioxide can induce growth in yeast cells, yet this possible explanation for the growth behaviour of cultures sharing headspace is never addressed.

We focused on sulfur-containing volatiles because the current understanding of sulfur metabolism in yeast suggested that the growth of *met17Δ* was limited by the availability of essential organosulfurs (OrgS). All other nutrients are provided in plenty in yeast minimal medium and should not have been limiting. While other compounds could in principle have served as signalling molecules to activate unknown OrgS synthesis pathways, our experiments immediately implicated sulfide: Firstly, sharing headspace with other OrgS auxotrophs led to longer lags in low-density *met17Δ* cultures (compare Fig 2C with left panel in Fig 2E), suggesting that lags were sensitive to the available amount of a volatile sulfur compound. Secondly, addition of sulfide increased the propensity of growth where other volatile compounds should not have been affected (Fig 2G). Finally, we have now included additional experimental evidence, showing that when sulfide is removed from the headspace of 96-well plates by inserting lead acetate paper in the gaps between the wells, the lag time of low-density cultures increased considerably (Fig 2F). Altogether, we find that sulfide is sufficient to explain the density-dependent growth of *met17Δ.*

The authors note a background strain-dependent difference in met17_delta phenotype. I would appreciate a discussion of why this could be, especially since RM11 is a wine isolate and the importance of sulfide content on wine quality.

The strain-dependent differences in the behaviour of *met17Δ* possibly result from a combination of higher sulfide production and higher *HSU1* activity in RM11 (L232-L239). It is however unclear whether this particular activity of *HSU1* has any relevance in wildtype yeast (when *MET17* is functional) since *MET17* is likely to be more efficient at metabolizing H_2S. Nevertheless, we now mention this observation, alongside the high degree of strain-to-strain sequence variability in *HSU1* (S20 Fig), in our Discussion section “Possible functions of *HSU1* in sulfur metabolism” (L631-L686). This section has now been updated to accommodate new competition assay results. We speculate on the catalytic role of *HSU1* and how it may contribute to the production of volatile compounds and connect this to application in winemaking.

The manuscript would benefit from a clearer integration of the proposed mathematical model and the observed phenotypes. Suggestions include validating the model's predictive capabilities through new experiments and/or using the model to explain the mechanisms behind the phenotypes.

We apologize that the integration of the model and the observed phenotypes did not come across clearly in our writing. In fact, our model is shaped from experimental findings and subsequently reproduces the two key features of density-dependent growth of *met17Δ*—longer lags and stochastic lag times at lower cell densities. We have now added a paragraph to the Discussion section “Considerations for quantitatively investigating volatile-mediated microbial interactions” explicitly explaining the integration and interplay of the model and experiments (L585-L603).

I would appreciate a clearer distinction between the Results and Discussion sections. For example, L230-240 and L435-466 within Results would be better fit for Discussion.

We apologize if the reviewer found this irksome. Both these cases were not mistakes, but conscious narrative choices. We only did this in instances where subsequent experiments in the Results section needed to be motivated by our interpretation of published literature. However, we realize that these may interrupt the flow of the reading, so we have redistributed the information to cut down the Discussion-like material in both those instances. The information on OAS sulfhydrylases from other yeast species has been moved to the Introduction (L96-L99). The explanation of why parameters from biochemical studies cannot be used in our model has been moved to the Methods (L911-L923). We felt this point was largely of technical interest and would not belong in the Discussion.

The manuscript would benefit from proofreading, especially figure captions and references, strain number corresponding to genotype and background, and Table S1, incuding the exact parent strain(s) of the constructed as well as clear references.

We apologize for the errors that escaped our previous proofreading. We have corrected errors in the captions and figure references. We have double-checked that strain numbers are correctly cited in the figure legends and their genotypes and backgrounds are detailed in Table S1. We have included references wherever applicable in Table S1; however, most of the strains were generated in our lab and are unpublished. Our strains have been generated through multiple rounds of manipulation over many generations. Providing parental genotypes for each strain will not be feasible as every parent from every preceding generation will then need to be added to the strain list. We have however described the generation of many of our strains in the Methods section “Yeast strains” in considerable detail. We have also added a note in the Strain List that our RM11 strains are derived from ancestors procured from the Gottschling lab.
